# A high content (30%) stable doxycycline hyclate drinking-water solution for broilers: preparation, stability, and bioequivalence

**DOI:** 10.3389/fvets.2025.1740059

**Published:** 2026-01-07

**Authors:** Ziyao Liu, Xiaojun Jiao, Linhua Xie, Wenge Ren, Linyi Lv, Juan Du, Xianhui Huang

**Affiliations:** Guangdong Key Laboratory for Veterinary Drug Development and Safety Evaluation, College of Veterinary Medicine, South China Agricultural University, Guangzhou, China

**Keywords:** bioequivalence, broilers, doxycycline hyclate solution, pharmacokinetics, stability study

## Abstract

**Introduction:**

Doxycycline is a semi-synthetic tetracycline antibiotic with broad-spectrum antimicrobial activity. Under intensive animal husbandry, commercially available doxycycline drinking-water formulations often show several drawbacks in solubility, stability and homogeneous distribution under farm conditions. To overcome these limitations, we developed a high content (30%, w/v), pilot-scale, stable doxycycline hyclate solution (DOX·HCl solution) specifically designed for drinking-water administration. A comprehensive quality assessment of this system was performed, followed by a bioequivalence evaluation of its application in broilers.

**Method:**

Prescription selection through high-temperature testing was combined with preparation process study via single-factor screening to prepare DOX·HCl solution. Quality assessment included assay of DOX, the dilutability and dilution stability testing, evaluation of mixed performance, and the stability studies. Bioequivalence study was conducted in a randomized, parallel, and blinded design to compare the DOX·HCl solution with two commercially available doxycycline hyclate drinking-water products.

**Results:**

Three pilot-scale batches of the 30% DOX·HCl solution (mean content 100.14 ± 0.37%) were successfully manufactured under controlled conditions of 400 rpm stirring and 60 °C. These pilot samples remained stable in appearance and content when diluted with water for up to 24 h. Affecting factor testing, accelerated testing and long-term testing all showed good stability, with an expiration date exceeding 24 months. In broilers, the DOX·HCl solution achieved a T_max_ of 3.19 ± 0.86 h, *t*_1/2_ of 6.47 ± 2.77 h, C_max_ of 7.69 ± 2.27 μg/mL, and AUC_0-t_ of 79.48 ± 23.88 h·μg/mL. There was no significant difference (*p* > 0.05) in the main pharmacokinetic parameters among the experimental broiler groups, and 90% CI values were within 80–125%, indicating that the 30% DOX·HCl solution was bioequivalent to the commercially available drinking-water formulations.

**Conclusion:**

The 30% DOX·HCl solution represents a high-content, industrially scalable drinking-water concentrate that combines long-term physicochemical stability with bioequivalence to existing products. This formulation offers a more convenient and highly efficient reliable option that could be a potential alternative to commercial products for mixed drinking-water medication of livestock and poultry, particularly under high-density, centralized management conditions.

## Graphical abstract

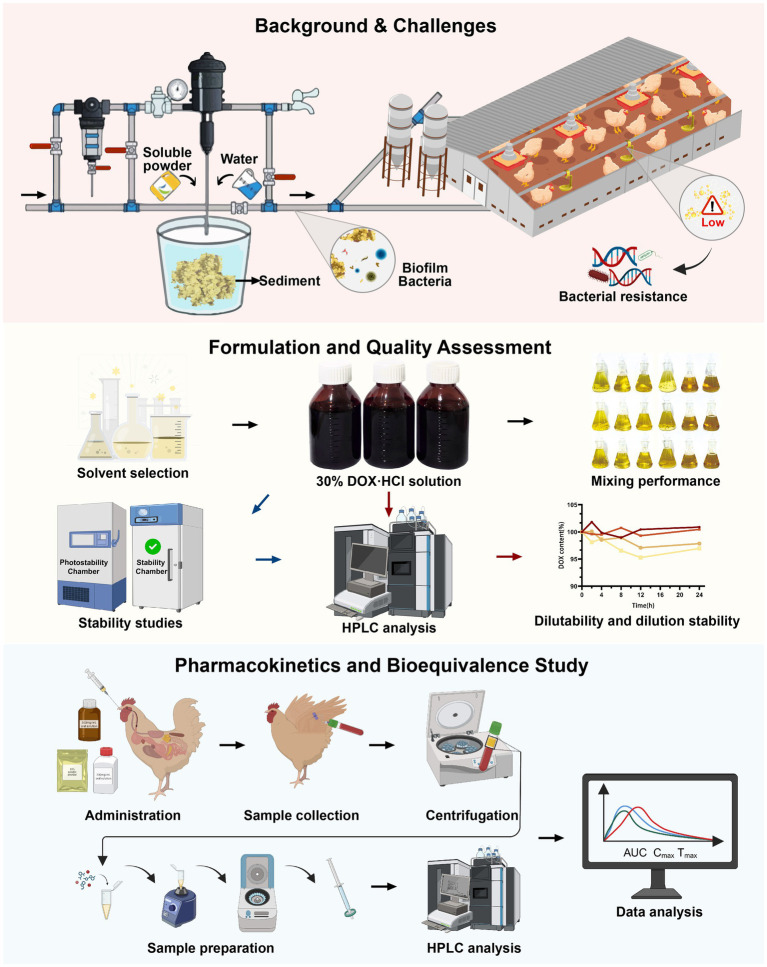

## 1 Introduction

Within the development of intensive animal husbandry, bacterial respiratory diseases pose a significant threat, resulting in substantial annual economic losses. Doxycycline, a semi-synthetic tetracycline, has greater lipophilicity than older tetracyclines, which confers advantages such as high oral bioavailability and a wide volume of distribution in animals (1, 2). Like other tetracyclines, doxycycline exhibits a wide range of activity against both G^+^ and G^−^, including avian cholera and colibacillosis (3, 4). The treatment of infections caused by *Mycoplasma gallisepticum* and *Mycoplasma synoviae* has also been found to be efficacious (5–8). Because of these properties, doxycycline is widely used in veterinary medicine, most commonly as solid dosage forms such as doxycycline hyclate granules and soluble powders, as well as oral solutions (9–11).

The administration of drugs via drinking water is a common method in livestock and poultry (particularly pigs and poultry) management due to its high compliance in diseased animals, suitability for group administration, and rapid drug absorption (12–14). A well-designed drinking water system ensures uniform drug distribution and reduces the risk of drug resistance (13–15). Currently, commercial doxycycline hyclate products for drinking water include soluble powder and oral solution (200 mg/mL) (16, 17). In mainland China, among legally marketed veterinary products containing doxycycline hyclate, drinking-water solution dosage forms are currently absent, and no imported registrations of foreign liquid solutions are in place. As result, poultry medication relies predominantly on soluble powders for water administration. However, the combination of high-density farming and centralized management places considerable demands on the solubility and stability of conventional doxycycline hyclate formulations. Soluble powder, when dissolved at ultra-high concentrations, is prone to issues such as sediment, uneven dissolution, and the formation of biofilms that can clog water lines (18). These problems may lead to insufficient drug concentrations at the clinical endpoint and increase the risk of antimicrobial resistance. This creates a clear, context-specific need for high-content, quality-controlled liquid concentrates designed for accurate dilution in poultry.

At present, overseas commercially available doxycycline hyclate oral solutions with a concentration of 100 mg/mL or 200 mg/mL can partially address the aforementioned issues (17, 19). However, for practical reasons (construction of poultry houses, handling large amounts of poultry) and a single water source (often a water tank of 500–1,000 L), there is still a need for higher-strength preparations that can be conveniently used in large-volume water systems. Meanwhile, doxycycline solid formulations (The aforementioned) can be effectively isolated from oxygen, light, and moisture at room temperature, significantly delaying oxidation, epimerization, and maintaining stability. In contrast, liquid formulations require stricter storage conditions, including refrigeration below 4 °C, to prevent high-temperature, oxygen-induced degradation and cryogenic precipitation. These stringent requirements limit the development of liquid doxycycline hyclate formulations (20). Consequently, we aimed to develop a high content (30%, w/v) stable doxycycline hyclate drinking-water solution for broilers and to conduct a comprehensive quality and bioequivalence evaluation, and it will be compared with commercially available formulations sharing the same route of administration to provide a new clinical solution for centralized drinking-water administration.

## 2 Materials and methods

### 2.1 Materials

Doxycycline hyclate (Lot No.7092206026, purity 90.4%) was purchased from Shandong Guobang Pharmaceutical CO., Ltd. DOX standard (Lot No.7130485–202,104, purity 83.6%) and metacycline (Lot No. 130499–200,802, purity 88.5%) were purchased from the China Institute of Veterinary Drugs Control (Beijing, China). Chromatography grade methanol and acetonitrile were obtained from Thermo Fisher Scientific (China). Ammonium acetate, triethylamine, ammonia solution and hydrochloric acid were purchased from Guangzhou Chemical Reagent Factory (Guangzhou, China). Propylene glycol was purchased from Shanghai Aladdin Biochemical Technology Co., Ltd. (Shanghai, China). Water for injection was purchased from Richeng County Tongren Veterinary Medicine Co., Ltd. All other reagents were of analytical grade. The three batches of pilot samples were manufactured under commission by Tianjin Speerise Challenge Biotechnology Co., Ltd. (Tianjin, China), and the doxycycline hyclate soluble powder was also a product of the same company. DFV DOXIVET 200 mg/mL oral solution was manufactured by DIVASA-FARMAVIC, S. A. (Barcelona, Spain).

### 2.2 Preparation of 30% DOX·HCl solution

#### 2.2.1 Assay of DOX

The content of DOX in DOX·HCl solution was determined by High Performance Liquid Chromatography (HPLC; LC-20AT; Shimazu, Japan). The detection wavelength in HPLC was set at 280 nm, a XBridge C18 column [5 μm, 4.6 × 250 mm, Waters Corporation (Shanghai) Co. Ltd.] was used for separation at 35 °C. The mobile phase was acetate buffer [0.25 mol/L ammonium acetate solution-0.1 mol/L ethylenediaminetetraacetic acid disodium solution-triethylamine (100:10:1), adjusted to pH 8.8 with glacial acetic acid or ammonia solution]-acetonitrile (85:15, v/v) with a flowrate of 1.0 mL/min. The linear range of DOX was at 0.10–0.30 mg/mL. A sample evaluation was carried out employing an HPLC method validated for the determination of DOX and its related substances in solution. Test solutions were prepared by diluting samples with 0.01 mol/L HCl solution [as described in the Chinese Veterinary Pharmacopoeia (21)] to obtain DOX concentrations of 0.20 mg/mL, and 20 μL of each solution was injected in duplicate.

#### 2.2.2 Solvent selection

Following a preliminary experiment, dimethylformamide (DMF), PEG400, glycerol formal (GF), *α*-pyrrolidone (α-P) and propylene glycol (PG) were selected as potential solvents. The prescribed quantity of doxycycline hyclate was dissolved separately in each solvent and the solutions were subjected to high-temperature testing at 60 °C. Samples were collected on the 10th day. The determination of the appearance and content served as primary evaluation parameters; if necessary, the solvents could be combined, and then the related substances were determined as the differentiating indicator for evaluation.

#### 2.2.3 Preparation process study

Considering that the rotation speed was fixed in pilot production, the stirring speed was set at 400 rpm. Temperature was identified as the key process parameter for this solution system. Therefore, preparation temperatures of 50 °C, 60 °C, and 70 °C were evaluated. Taking appearance, content and related substances as indicators, the optimal preparation temperature was screened out.

#### 2.2.4 Preparation of DOX·HCl solution

The DOX·HCl solution was an oil solution consisting of doxycycline hyclate and an appropriate organic solvent screened in section 2.2.2. The preparation process involved heating a suitable volume of propylene glycol to the temperature screened in section 2.2.3, followed by the addition of the prescribed quantity of doxycycline hyclate. The mixture was stirred at 400 rpm while maintaining the temperature for 40 min, then filtered and packaged to obtain the final product.

#### 2.2.5 Dilutability and dilution stability

To investigate the dilatability of DOX·HCl solution prepared in this study at different ratios in clinical use scenarios, DOX·HCl solution was diluted with purified water at ratios of 1:10, 1:50, 1:100 and 1:1000, and the concentration was determined by HPLC. According to the livestock and poultry drinking water standards issued by the Chinese Ministry of Agriculture and Rural Affairs (22), simulated drinking water for livestock and poultry was prepared with a maximum total hardness of 1,500 mg/L (calculated as CaCO_3_) and a pH range of 6.5–8.5. Commercially available doxycycline hyclate soluble powder and DOX·HCl solution we prepared were diluted with the simulated water to a final administration concentration of 300 mg/L (calculated as doxycycline) and maintained at room temperature. The same dilution procedure was performed using water for injection as a control group. The clinical dilution stability of the two formulations was assessed using HPLC.

#### 2.2.6 Mixed performance evaluation

Commercially available 10% (w/w, A) and 50% (w/w, C) doxycycline hyclate soluble powders were purchased, and the 30% DOX·HCl solution (w/v, B) prepared in our study was included for comparison. Each formulation was mixed with 100 mL of water to achieve final doxycycline concentrations of 30 mg/mL and 50 mg/mL. The solutions were vigorously shaken for 30 s at 5-min intervals, and the mixing behaviors of A, B, and C were recorded.

### 2.3 Stability studies

To investigate the effects of DOX·HCl solution on temperature and bright light, it was placed at 40 °C for the high-temperature testing and at an illumination of 4,500 lx ± 500 lx for photostability testing, both of which were sampled on days 0, 5 and 10 for detection of DOX by HPLC. Three batches of pilot samples of DOX·HCl solution packaged in pharmaceutical grade high-density polyethylene (HDPE) plastic bottles were kept at 40 °C/75% relative humidity for 6 months for accelerated testing, and at 25 °C/60% relative humidity for 24 months for long-term testing. Then, the content of DOX was analyzed using HPLC (Section 2.2.1).

### 2.4 Bioequivalence study

#### 2.4.1 Experimental animals

One hundred and twenty 60-day-old healthy spotted-brown broilers with a mean weight of 2.15 ± 0.25 kg were procured from a commercial farm in the outskirts of Guangdong Zhicheng Food Co., Ltd. (Shaoguan, China). Before the experiment, the broilers were housed in wire cages within the animal facility, allowing for visual contact with one another to mitigate potential anxiety. Throughout the 1-week acclimatization phase, the ambient temperature was kept at 25 ± 2 °C, with a light–dark cycle of 16 and 8 h, respectively. Free access to water was ensured, and the broilers were fed antibiotic-free feed to prevent any interference from antibiotics in the experiment. Daily observations were conducted on the experimental subjects to monitor their feather condition, dietary intake, and behavioral patterns, ensuring their good health. Ethical approval for bioequivalence study in broilers was obtained from the Animal Ethics Committee of South China Agricultural University. All animals were housed and experiments conducted in strict accordance with the norms for the care and use of laboratory animals.

#### 2.4.2 Drug information

The test product 30% doxycycline hyclate solution (Lot No.202206001, 100 mL: 30 g), was the pilot sample. The reference product 1 DFV DOXIVET 200 mg/mL oral solution (Lot No: S-005, 1 L: 200 g) was purchased by DIVASA-FARMAVIC, S. A. (Barcelona, Spain). The reference product 2 10% doxycycline hyclate soluble powder (Lot No.202302001, 100 g: 10 g) was provided by Tianjin Speerise Challenge Biotechnology Co., Ltd. (Tianjin, China).

#### 2.4.3 Experimental design and sample collection

The current study was a parallel, randomized, single-blind design, with 108 broilers randomly assigned to either the test product group, the reference product 1 group or the reference product 2 group. The remaining broilers were used as blank sample sources and for methodological purposes. The broilers involved in the experiment underwent a 16-h fasting period before administration, followed by normal feeding 4 h post administration, with no restriction on drinking. Just before administration, the soluble powder and oral solution were prediluted with water for injection to facilitate gavage administration. Subsequently, doxycycline was administered via gavage at 20 mg/kg.b.w. Blood samples were collected from the brachial wing veins at 0 h (before dosing), 0.25, 0.5, 1, 1.5, 2, 3, 4, 5, 6, 9, 12, 16, 24, 30, and 36 h following administration. Approximately 1.5 mL of blood was collected at each specified time point and placed in sodium heparin tubes. Immediately after collection, the blood samples were placed on crushed ice and centrifuged at 4,000 rpm for 10 min at 4 °C to separate the plasma. The plasma samples were then stored at −20 °C until analysis.

#### 2.4.4 Sample preparation and analysis

Briefly, each sample (400 μL) containing the IS metacycline (2.5 μg/mL) was mixed with acetonitrile (790 μL), vortexed thoroughly for 2 min, and centrifuged at 12,000 rpm, 4 °C for 10 min. Subsequently, the supernatant was evaporated under a nitrogen stream at 40 °C. The resulting residue was dissolved in 200 μL 0.01 mol/L hydrochloric acid, filtered through a 0.22 μm nylon syringe filter, and injected 20 μL into the HPLC system for analysis.

The HPLC analysis conditions for doxycycline and internal standard (IS) metacycline in plasma were adapted from a previous study (21) and further optimized for this experiment. A similar liquid phase system and column were employed as described in section 2.2.1, while the detection wavelength was set at 350 nm to minimize interference from endogenous matrix components. The mobile phase consisted of acetate buffer-acetonitrile (83.5:16.5, v/v) at a flow rate of 1.0 mL/min.

#### 2.4.5 Validation of HPLC method

The selectivity, sensitivity, standard curve, accuracy, precision, stability, and dilution reliability of the proposed approach were verified following the “*Guiding Principles for the Validation of Quantitative Analysis Methods for Biological Samples*.”

To demonstrate the selectivity, chromatograms obtained from six batches of mixed blank chicken plasma, blank plasma spiked with DOX standard solutions and plasma samples after administration of DOX were compared. The limits of detection (LOD) and quantitation (LOQ) were established using drug-free matrix spiked with known concentrations of DOX (with IS), and the lowest concentration in the calibration curve was required to yield a signal-to-noise (S/N) ratio of ≥3 and ≥10, respectively.

A standard stock solution of doxycycline (2 mg/mL) was prepared by dissolving the reference substance in methanol and stored at −20 °C away from light. The stock solution was subsequently diluted in 0.01 mol/L HCl to produce a range of working solutions with concentrations spanning from 10 to 400 μg/mL. Subsequently, these working solutions were added to blank plasma samples to establish the calibration curve, covering DOX concentrations from 0.25 to 10 μg/mL. Least-squares linear regression was used to fit the area ratio to the concentration (A = 1/C^2^, A: absorption peak area ratio of target compound to internal standard, C: concentration of DOX).

To assess the precision and accuracy of this assay method, 3 distinct spiked concentrations of DOX QC samples (LQC 0.75 μg/mL, MQC 4 μg/mL, and HQC 8 μg/mL) were prepared with six identical parallel samples for each concentration. The intraday precision and accuracy were calculated for a single day of analysis, while the interday precision and accuracy were calculated for three consecutive days.

The stability of DOX was evaluated by preparing six parallel DOX QC samples (LQC and HQC) repeatedly through the sample preparation method under the following three storage conditions: (1) untreated samples were placed at room temperature for 5 h; (2) samples were left in the autosampler (26 °C) for 24 h; (3) samples were subjected to freeze–thaw cycles for three times; (4) samples were stored in a − 20 °C refrigerator for 3 months.

In instances where the concentration of DOX exceeded the maximum value of the calibration curve, samples were diluted with 0.01 M HCl solution to ensure accurate quantification. The reliability of dilution was evaluated by preparing samples at a concentration exceeding the upper limit of the standard curve (25 μg/mL), and then diluting them with blank plasma matrix.

#### 2.4.6 Pharmacokinetics analysis

Plasma samples for each sampling point from 108 broilers were analyzed for DOX concentration by HPLC. Then the pharmacokinetic parameters of DOX for each broiler were obtained by using Phoenix 64 WinNonlin 8.1 software (Certara, New Jersey, USA) for noncompartmental analysis (NCA). The main pharmacokinetic parameters were compared between the three groups. Also, the three pharmacokinetic parameters of *C*_max_, AUC_0-t_, AUC_0-∞_were used to analyze the bioequivalence of the three preparations.

#### 2.4.7 Bioequivalence analysis

An analysis of variance (ANOVA) of the main pharmacokinetic parameters (*C*_max_, AUC_0-t_, AUC_0-∞_) between three groups was performed using SPSS commercial software (version 26.0; IBM, Armonk, NY). Meanwhile, the bioequivalence was evaluated using Phoenix 64 WinNonlin 8.1 (Certara, New Jersey, USA). Bioequivalence was assessed according to standard acceptance criteria: *C*_max_, AUC_0-t_ and AUC_0-∞_ were log-transformed and analyzed by ANOVA and two-sided t-test with statistical significance defined as *p* < 0.05. If the 90% confidence intervals of the geometric mean ratios (GMRs) for Ln (*C*_max_), Ln (AUC_0-t_) and Ln (AUC_0-∞_) were contained within 80–125%, the test product was considered bioequivalent to the reference product.

## 3 Results

### 3.1 Preparation of 30% DOX·HCl solution

#### 3.1.1 Solvent selection results

All solvents except PEG400 dissolved the prescription quantity of doxycycline. After 10 days at 60 °C, the DMF and glycerol formal formulations had turned darken brown to brown clear liquids, whereas the *α*-pyrrolidone and propylene glycol formulations remained brown clear liquids. The decrease in DOX content followed the order DMF>GF>5%>α-P>PG.

**Figure 1:**
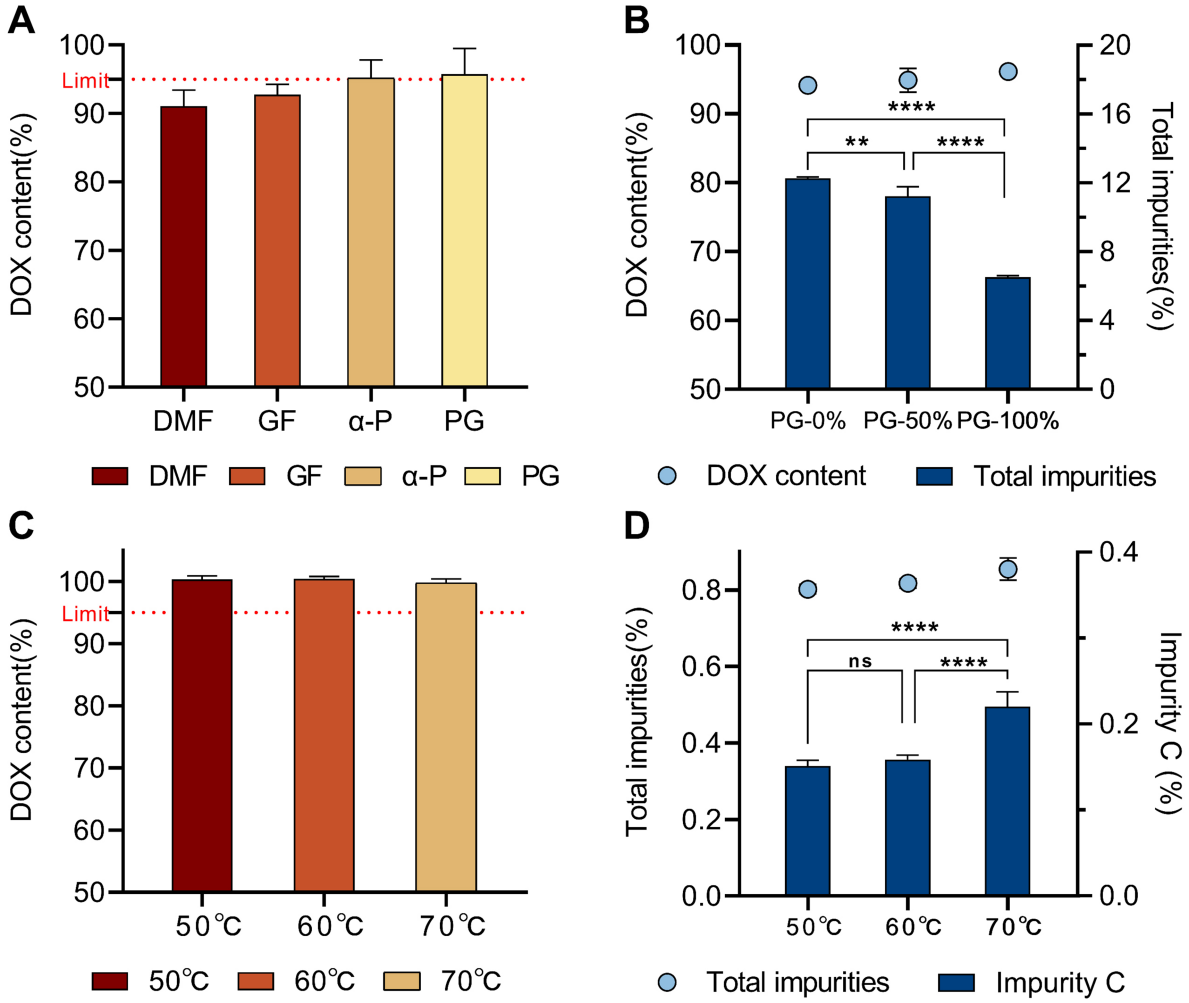
Results of solvent selection and process optimization for the 30% DOX·HCl solution. **(A)** DOX content in different single-solvent systems (DMF, PEG400, glycerol formal, *α*-pyrrolidone and propylene glycol)after storage at 60 °C for 10 days; **(B)** Effect of increasing propylene glycol (PG) proportion (0, 50%, and 100%) on DOX content and total impurities (60 °C, 10 days); **(C)** Effect of different preparation processes (50 °C, 60 °C, 70 °C) on DOX content; **(D)** Corresponding changes in the main degradation product 4-EDOX and total impurities under these process conditions. Significance: ns, not significant (*p* ≥ 0.05); *p < 0.05; **p < 0.01; ***p < 0.001; ****p < 0.0001.

Furthermore, taking α-Pyrrolidone, which exhibited the second-best performance in terms of appearance and content, as the solvent capable of meeting the basic solubility requirements, propylene glycol was incorporated at ratios of 0, 50 and 100%. Under the same stress conditions (60 °C, 10 days), appearance, DOX content and related substances were monitored. As the proportion of propylene glycol increased from 0 to 100%, both the color change and content loss were reduced, while the levels of related substances showed a clear downward trend. Taken together (Figures 1A,B), these results demonstrate that the use of propylene glycol alone as a solvent is superior to its combination with other solvents. Therefore, propylene glycol was selected as the solvent for the DOX·HCl solution to improve both the solubility and stability of DOX.

#### 3.1.2 Preparation process of DOX·HCl solution

In the prescription study, it was found that doxycycline hyclate dissolved incompletely when the solution was prepared at room temperature, indicating that the preparation of the product required certain heating conditions. Under the stirring speed of 400 rpm, the dissolution of doxycycline hyclate accelerated and was completely dissolved as the preparation temperature increased from 50 °C to 70 °C. The appearance, content and related substances of the system were all qualified, but there was a certain deepening of color at 70 °C. Combined with Figures 1C,D, the impurity C (4-epidoxycycline, 4-EDOX) appeared to be significantly increased at 70 °C compared with 50 °C and 60 °C. Considering the actual production conditions and costs, the stirring speed of 400 rpm and the preparation temperature of 60 °C were determined as the optimal preparation process.

#### 3.1.3 Assay of DOX

**Figure 2:**
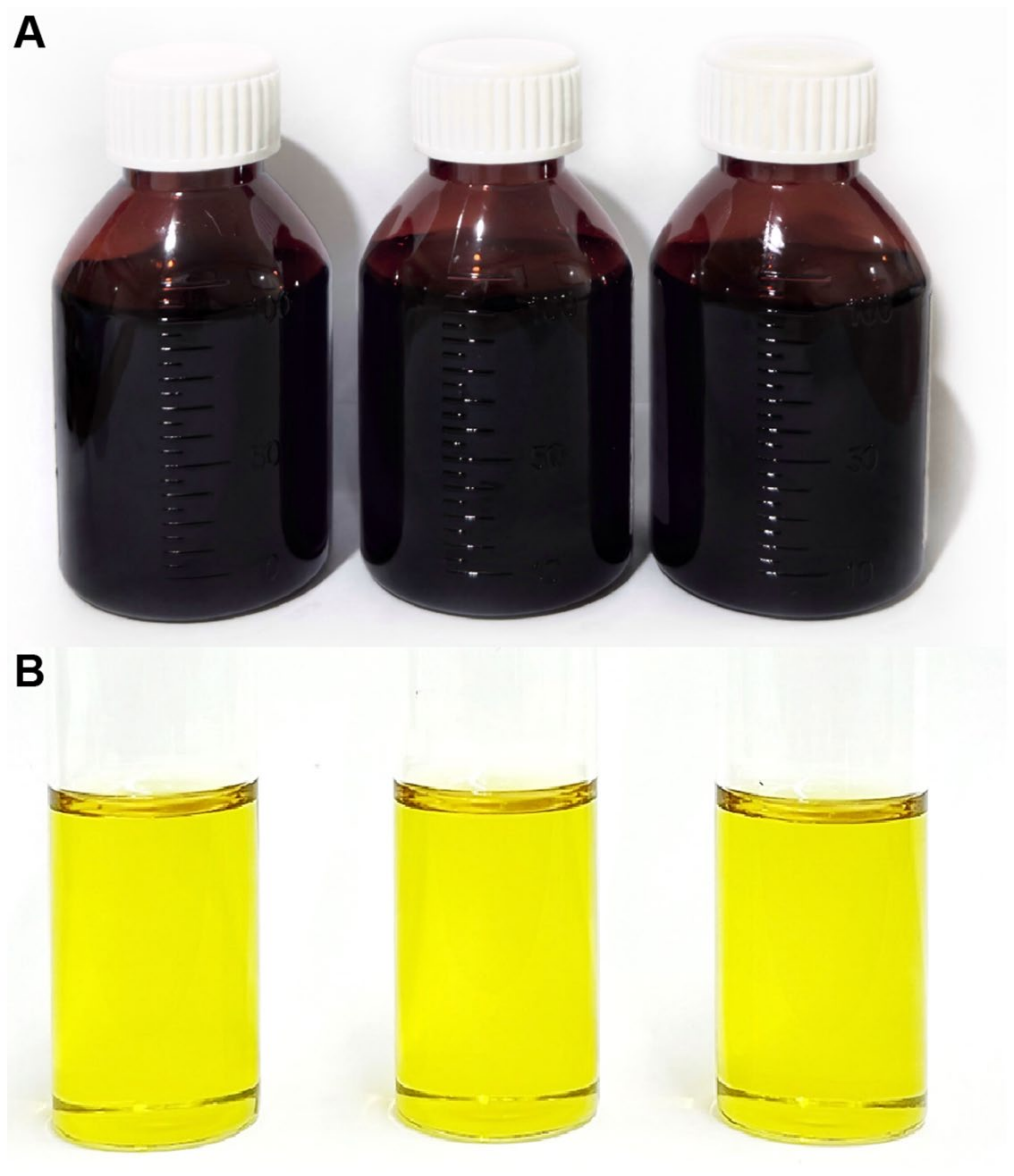
Representative images of 30% DOX·HCl solution. **(A)** Three pilot-scale batches filled into pharmaceutical-grade HDPE bottles; **(B)** Three laboratory-scale batches prepared under the same laboratory conditions.

Assay of DOX showed that the prepared DOX·HCl solution had a content specification of 30.04 ± 0.11 g/100 mL/bottle (Figure 2), which met the general rule.

#### 3.1.4 Dilutability and dilution stability

According to the HPLC results, all DOX·HCl solution samples diluted with water for injection at ratios of 1:10, 1:50, 1:100 and 1:1000 had DOX contents above 98.50%, meeting the predefined acceptance criteria (Table 1).

**Table 1:** Content of the solution after different proportions of dilution.

Dilution ratio	Content/%	Mean ± SD/%
1:10	98.50 ± 1.17	100.03 ± 1.50
1:50	100.90 ± 1.29	
1:100	99.04 ± 0.97	
1:1000	101.66 ± 0.48	

**Figure 3:**
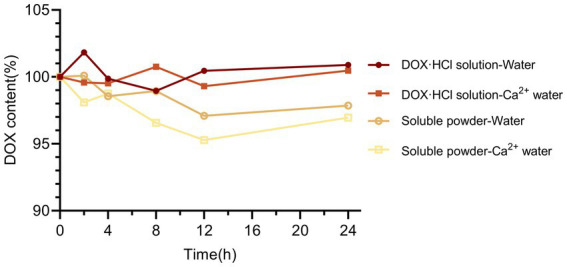
Stability of DOX·HCl solution and Doxycycline hyclate soluble powder at room temperature for 24 h after dilution in livestock drinking water and water for injection to the final administration concentration.

After dilution with water for injection and simulated water to the final administration concentration of 300 mg/L (calculated as doxycycline), the doxycycline hyclate soluble powder showed a slight decrease in content after 24 h at room temperature. In contrast, the DOX·HCl solution remained stable under both dilution conditions (Figure 3), indicating a good dilution stability in clinically relevant water matrices.

#### 3.1.5 Mixed performance evaluation

**Figure 4:**
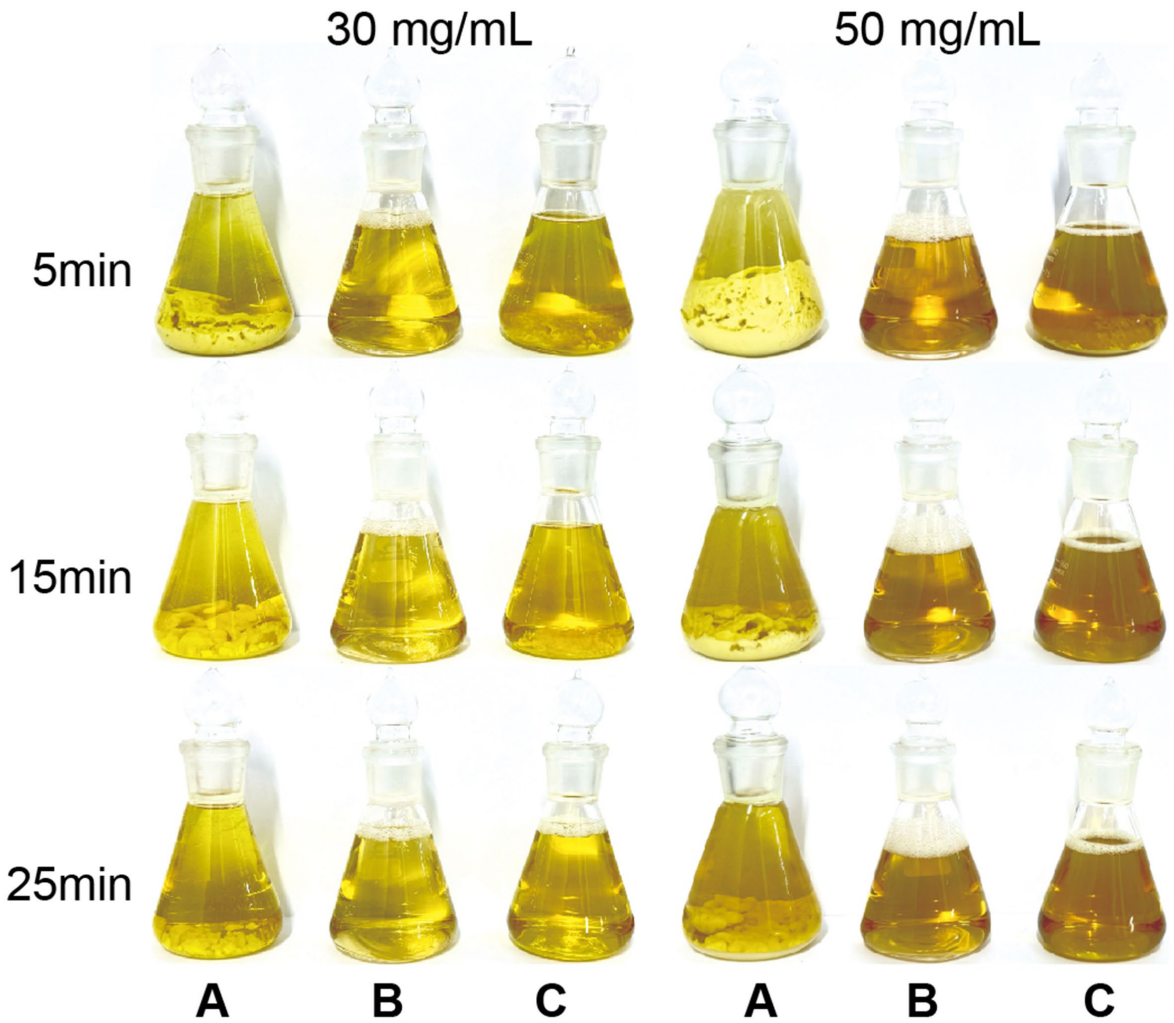
Mixing performance of different doxycycline formulations at high concentrations. Commercial 10% **(A)** and 50% **(C)** doxycycline hyclate soluble powders and the 30% DOX·HCl solution **(B)** were dispersed in 100 mL of water to obtain final doxycycline concentrations of 30 mg/mL and 50 mg/mL. Photographs show the appearance at 5, 15 and 25 min, illustrating that the DOX·HCl solution **(B)** forms a clear homogeneous solution more rapidly, whereas the soluble powders **(A,C)** exhibit persistent clumps/precipitates, especially at 50 mg/mL.

When mixed with water, the 10% doxycycline hyclate soluble powder (A) initially dissolved in a small amount, but rapidly agglomerated into a mass of wet precipitate with poor wettability and was difficult to disperse. The DOX·HCl solution (B) spread rapidly on the water surface, forming a clear liquid layer and then dissolving homogeneously without visible insoluble material. In contrast, the 50% doxycycline hyclate soluble powder (C) was only partially soluble and produced persistent insoluble precipitates that hindered complete dissolution. By 35 min, all three products were fully dissolved and appeared homogeneous and clear. Overall, the DOX·HCl solution showed markedly better initial mixing behavior than the two soluble powder products (Figure 4).

### 3.2 Stability studies

**Figure 5:**
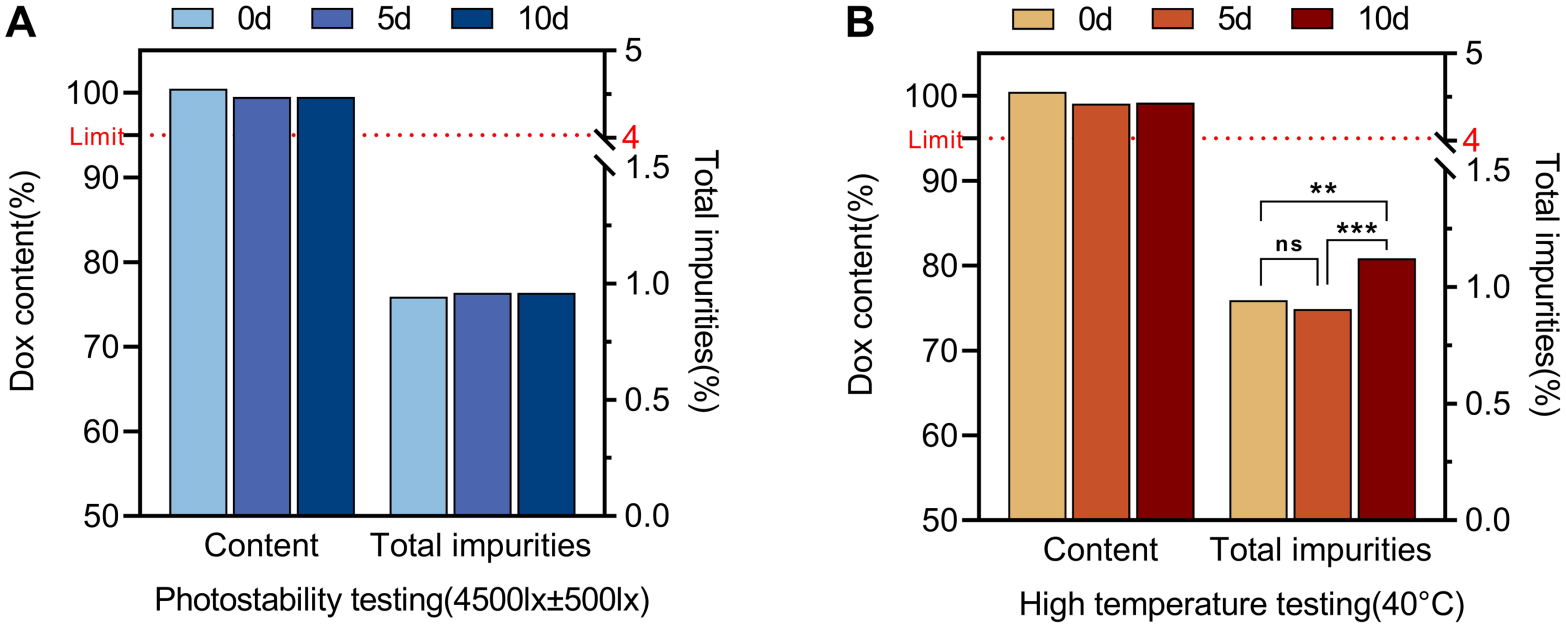
The affecting factor testing results of DOX·HCl solution. **(A)** Changes in DOX content and major degradation products during photostability testing at 4,500 ± 500 lx for 10 days. **(B)** Changes in DOX content, 4-EDOX and total related substances during high-temperature testing at 40 °C for 10 days. **p < 0.01; ***p < 0.001.

**Figure 6:**
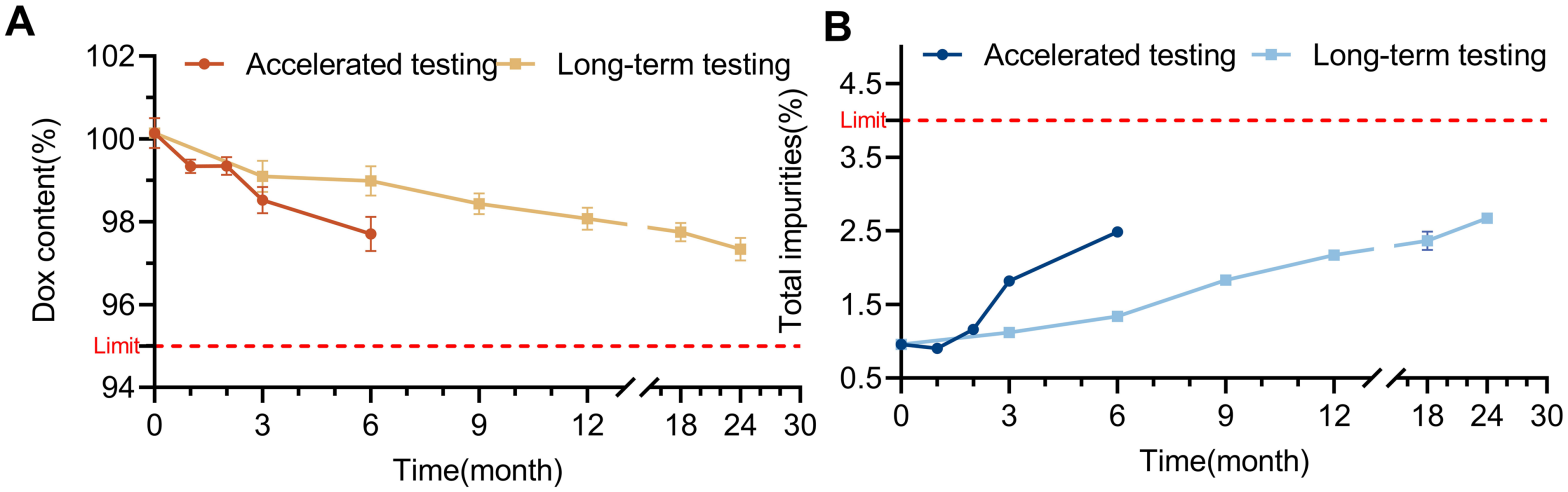
Stability of the 30% DOX·HCl solution. **(A)** DOX content and total related substances during accelerated testing at 40 °C/75% relative humidity for up to 6 months. **(B)** DOX content and total related substances during long-term testing at 25 °C/60% relative humidity for up to 24 months.

In the affecting factor testing (high-temperature testing and photostability testing), no significant change in DOX content was observed in the solution over 10 days. Although the level of 4-epidoxycycline (4-EDOX) increased significantly with time, the total impurities increased noticeably only at day 10 (Figure 5). Similar trends were observed in accelerated testing and long-term testing. When the DOX-HCl solution was stored at 25 °C/60% relative humidity for 24 months and 40 °C/75% relative humidity for 6 months, the DOX content showed only a minor decrease and related substances increased slightly (Figure 6). Indicating that the DOX·HCl solution possessed favorable stability characteristics.

### 3.3 Bioequivalence study

#### 3.3.1 Validation of HPLC method

**Figure 7:**
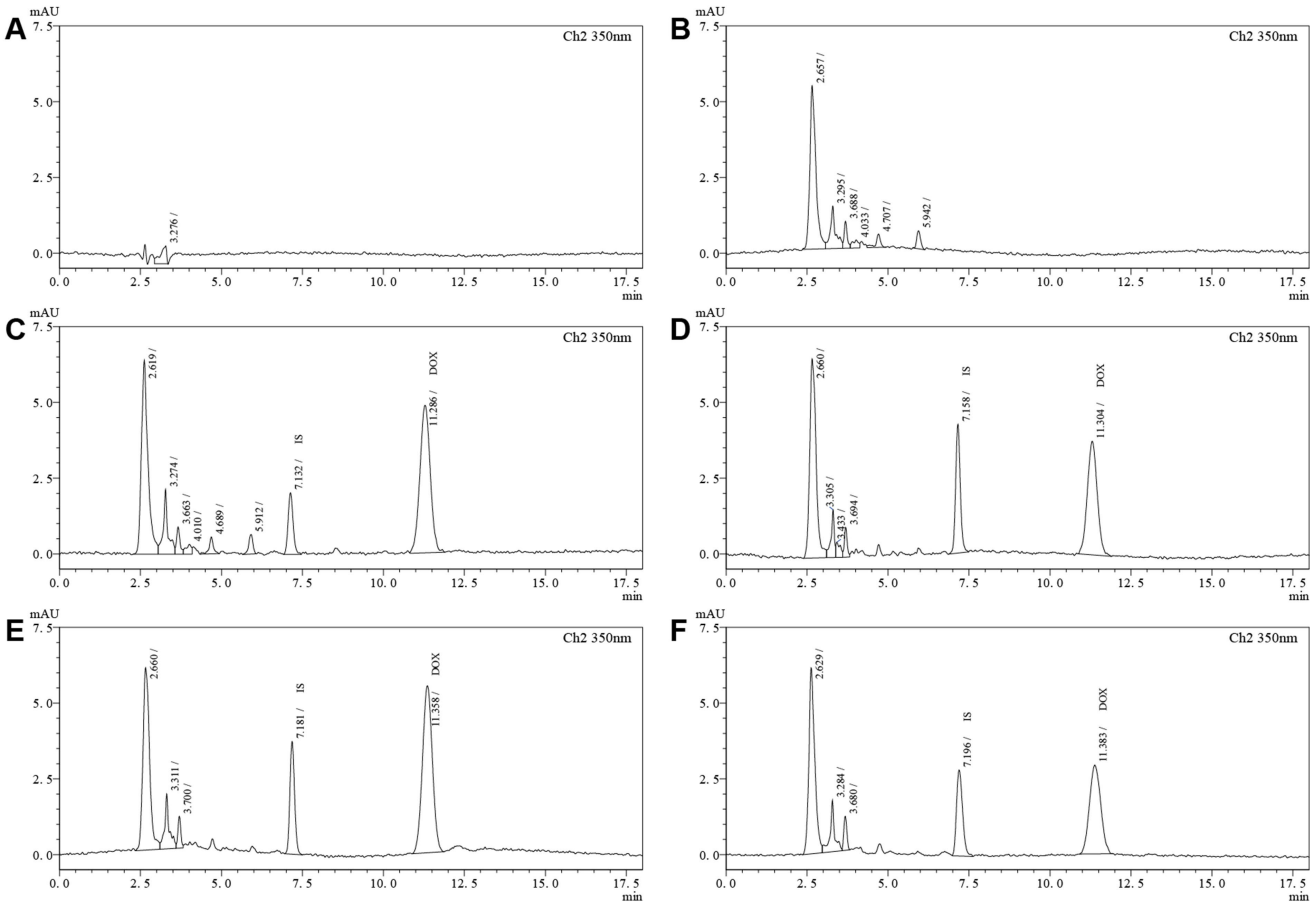
The specificity chromatograms of the HPLC method for determination of DOX in chicken plasma. **(A)** Chromatogram of diluent (0.01 mol/L HCl); **(B)** Blank chicken plasma; **(C)** Blank chicken plasma spiked with DOX standard (8.22 μg/mL) and IS (2.50 μg/mL); **(D)** Chicken plasma after administration of the test product (3.08 μg/mL); **(E)** Chicken plasma after administration of the reference product 1 (5.18 μg/mL); **(F)** Chicken plasma after administration of the reference product 2 (3.13 μg/mL).

As illustrated in Figure 7, the current extraction method was characterized by its simplicity and efficiency, coupled with a complete and effective separation of DOX and IS. DOX had a retention time of approximately 11.30 min, compared with 7.20 min for the IS. The peaks of DOX and IS showed a good peak shape and separation, with negligible interference from the endogenous plasma components and good specificity.

The LOD was 0.10 μg/mL and the LOQ was 0.25 μg/mL. Moreover, by adding known concentrations of DOX and metacycline to blank plasma samples, the linearity and reproducibility were excellent within the linear range of 0.25–10 μg/mL, regression correlation coefficient (r^2^) exceeding 0.99 (Table 2).

**Table 2:** Standard curve and correlation coefficient of DOX in chicken plasma.

Batch	Linear range	Regression equation	Correlation coefficient
1	0.25–10 μg/mL	A = 1.6095C + 0.0243	0.9947
2		A = 1.9213C-0.0294	0.9983
3		A = 1.7940C + 0.0246	0.9996

The average extraction recoveries of 0.75 μg/mL, 4 μg/mL and 8 μg/mL of chicken plasma were 90.55, 102.31, and 91.44%, respectively, and 95.97% for metacycline. As shown in Table 3, the intra-day and inter-day accuracy and precision for doxycycline at the LOQ concentration were within ±20%, while those for the three other concentrations (LQC, MQC, HQC) were within ±15%, meeting predefined acceptance criteria.

**Table 3:** The accuracy and precision of doxycycline in chicken plasma samples (Mean ± SD, *n* = 6).

Labeled values	Batch	Intra-day		Inter-day	
		Recovery/%	CV/%	Recovery/%	CV/%
0.25 μg/mL	1	95.47 ± 6.39	6.70	96.2 ± 9.77	10.16
	2	100.47 ± 14.06	13.99		
	3	92.67 ± 6.99	7.45		
0.75 μg/mL	1	107.04 ± 6.70	6.26	107.30 ± 5.83	5.43
	2	109.76 ± 6.69	6.10		
	3	105.09 ± 3.55	3.37		
4 μg/mL	1	110.50 ± 2.84	2.57	104.65 ± 5.36	5.13
	2	98.80 ± 2.22	2.24		
	3	104.64 ± 1.61	1.54		
8 μg/mL	1	105.72 ± 4.10	3.87	101.49 ± 5.46	5.38
	2	95.35 ± 2.01	2.11		
	3	103.40 ± 3.05	2.95		

The stability of the samples was investigated and analyzed by using two different concentrations, LQC 0.75 and HQC 8 micrograms/milliliter. The results show that it was stable under the four storage conditions listed in Supplementary Table 1. The average value of each measured concentration met the labeled values within ±15%. Following a 5-fold dilution of 25 μg/mL using a blank plasma matrix processed by the sample preparation resulted in an accuracy and CV% of 97.11 ± 5.83 and 6.00%, respectively, demonstrating the reliability of the dilution method.

Based on all the above results, the methodology validation results met the assay requirements.

#### 3.3.2 Pharmacokinetics analysis

**Figure 8:**
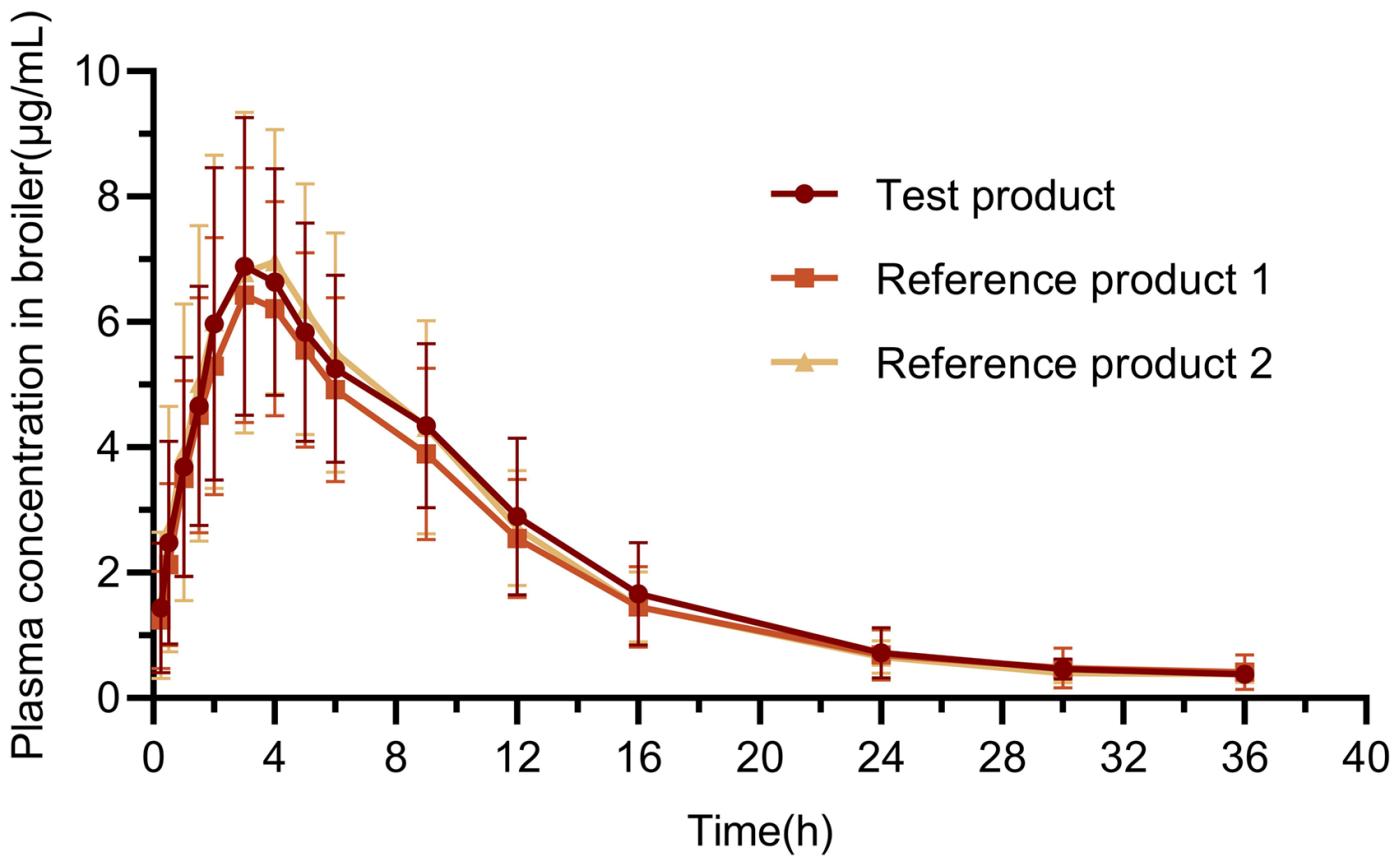
The mean ± SD plasma concentration-time profiles in broilers. The test product and reference product were administered at a single oral dose of 20 mg/kg.b.w (*n* = 36 for each product).

Throughout the experiment, all broilers exhibited normal eating habits. Following drug administration via both routes, the broilers did not display any adverse reactions such as vomiting, diarrhea, or depression. Figure 8 shows the plasma concentration-time profiles of oral administration of test and reference products, the commercial oral solution and powder at the equivalent dose of 20 mg/kg.b.w to broilers. Additionally, Table 4 shows the corresponding pharmacokinetic parameters. For the test product, T_max_ was 3.19 ± 0.86 h, *t*_1/2_ was 6.47 ± 2.77 h, and C_max_ was 7.69 ± 2.27 μg/mL. For the reference product 1, T_max_ was 3.26 ± 0.87 h, *t*_1/2_ was 5.94 ± 1.94 h, and C_max_ was 6.99 ± 1.86 μg/mL. The T_max_ of the reference product 2 was 3.47 ± 1.00 h, *t*_1/2_ was 6.16 ± 1.80 h, and C_max_ was 7.85 ± 2.45 μg/mL.

**Table 4:** Comparison of main pharmacokinetic parameters between the test product, reference product 1 and reference product 2 after 20 mg/kg oral administration in broilers (*n* = 36 for each product).

PK parameters	Units	Test product	Reference product 1	Reference product 2
*t*_1/2_	h	6.47 ± 2.77	5.94 ± 1.94	6.16 ± 1.80
T_max_	h	3.19 ± 0.86	3.26 ± 0.87	3.47 ± 1.00
C_max_	μg/mL	7.689 ± 2.27	6.994 ± 1.86	7.854 ± 2.45
AUC_0-t_	h·μg/mL	79.48 ± 23.88	72.49 ± 22.00	78.41 ± 24.61
AUC_0-∞_	h·μg/mL	82.21 ± 24.44	75.07 ± 23.44	80.84 ± 25.17

PK parameters are expressed as mean ± SD; *t*_1/2_: half-life; C_max_: maximum plasma concentration; T_max_: time to reach C_max_; AUC_0-t_: the area under the concentration-time curve from zero to defining time; AUC_0-∞_: area under the plasma concentration-time curve from zero to infinity.

#### 3.3.3 Bioequivalence analysis

No adverse events were found or reported in the test and reference groups throughout the whole study. The results of the bilateral two-sided *t*-test of the pharmacokinetic parameters obtained after logarithmic transformation are shown in Supplementary Table 2. The upper and lower limits of the calculated confidence intervals are presented in Table 5. There was no significant difference (*p* > 0.05) in the main pharmacokinetic parameters C_max_, AUC_0 − t_, and AUC_0-∞_ of DOX between the test and reference products after a single oral administration. Based on comparison of pharmacokinetic parameters, statistical analysis and the predefined acceptance criteria, the test product and the two reference products were concluded to be bioequivalent.

**Table 5:** Variance analysis of pharmacokinetic parameters and upper and lower limits of the calculated 90% CI.

Items	Parameters	*P*	90% confidence interval	
			CI_90_Lower	CI_90_Upper
Test product and reference product 1	Ln(C_max_)	0.211	97.74%	122.31%
	Ln(AUC_0-t_)	0.220	96.89%	123.90%
	Ln(AUC_0-∞_)	0.206	97.06%	124.25%
Test product and reference product 2	Ln(C_max_)	0.856	87.32%	111.60%
	Ln(AUC_0-t_)	0.837	89.64%	115.03%
	Ln(AUC_0-∞_)	0.799	90.10%	115.15%

## 4 Discussion

We successfully developed a doxycycline hyclate solution with a high content specification of 30% (w/v), which represents the current upper limit for DOX concentration in an oral liquid formulation suitable for drinking-water administration. Although commercially available doxycycline hyclate soluble powders already reach 50% (w/w), further increasing the content of oral solutions is challenging because of solubility limitations and stability problems. Many attempts have been made to prepare higher content DOX oral solutions, but these efforts were unsuccessful in our preliminary work (data not shown). Absolute ethanol has been proposed as a “water-inactivating” agent since it is known to form stable mixtures (azeotropes) with water (23, 24). However, when different proportions of ethanol (5%, 10% and 20%, v/v) were added in our preliminary formulations, all test solutions precipitated, likely due to the poor solubility of high-dose DOX in ethanol and the resulting instability of the whole solution system (11).

Two major challenges must be considered when developing a DOX·HCl solution for oral use: the stability of DOX in the concentrate itself and its stability after administration via drinking water. Stability is a critical concern since excessive degradation may lead to subtherapeutic dosages, which can potentiate the selection of resistant pathogenic strains in the long term, therefore leading to treatment failure and economic loss (12, 25, 26).

The key factor in this study was the choice of solvent system. DOX dissolved effectively in all selected solvent systems except PEG400. At the time of preparation, the formulations appeared clear and yellow to yellowish-brown, which is quite logical since DOX is a yellow powder. However, although some degree of color deepening could be observed in all the formulations during the study period, formulations DMF and glycerol formal darkened to a brown color upon storage. Although darkening can be caused by small amounts, it was considered as a signal of the presence of degradation products and therefore of DOX deterioration (27), which was precisely verified in the subsequent stability study.

Doxycycline hyclate is produced by the fermentation of oxytetracycline. During this process, the co-synthetic product 2-acetyl-2-deacetyldoxycycline (impurity F) was simultaneously generated (27). In our study, we observed that impurity F decreased with increasing time and temperature until it eventually disappeared. This phenomenon explains why 4-EDOX increased significantly over time, whereas the total impurity content increased markedly only at day 10 in high-temperature testing, and no similar trend was observed in the photostability testing. These findings indicate that the DOX·HCl solution is more sensitive to temperature but exhibits relatively stable performance under light exposure (28, 29). However, the solvent and other parameters such as pH, temperature, Oxygen or the presence of cations have been suggested to influence keto-enol tautomerism, the epimerisation of the dimethylamino group in C4, facilitating the formation of typical degradation products (including epimers) of doxycycline, leading to drug inactivation (30–33).

Because drinking water quality parameters may have unknown effects on the pharmacokinetic behavior of doxycycline (34), and to ensure the optimal performance of the DOX·HCl solution in practical clinical applications, a comparative evaluation of dilution stability and mixing performance was conducted between the DOX·HCl solution and doxycycline hyclate soluble powder. This study aimed to provide a direct comparison with the current drinking water administration method and to address the mixing kinetics and solubility issues highlighted by Vervaet et al. (18). In all solvent systems, propylene glycol was selected as the primary solvent due to its intermediate dielectric constant (*e* = 32) falls between that of water (*e* = 80) and those of non-polar solvents (*e* < 2) (35). This characteristic makes it a suitable candidate for dissolving lipophilic compounds, such as DOX, while maintaining miscibility with water (36, 37). Meanwhile, in the composition of the formulation prescription, it was not difficult to find the presence of propylene glycol, which also indicated that it had relatively high safety and biocompatibility (38, 39).

**Figure 9:**
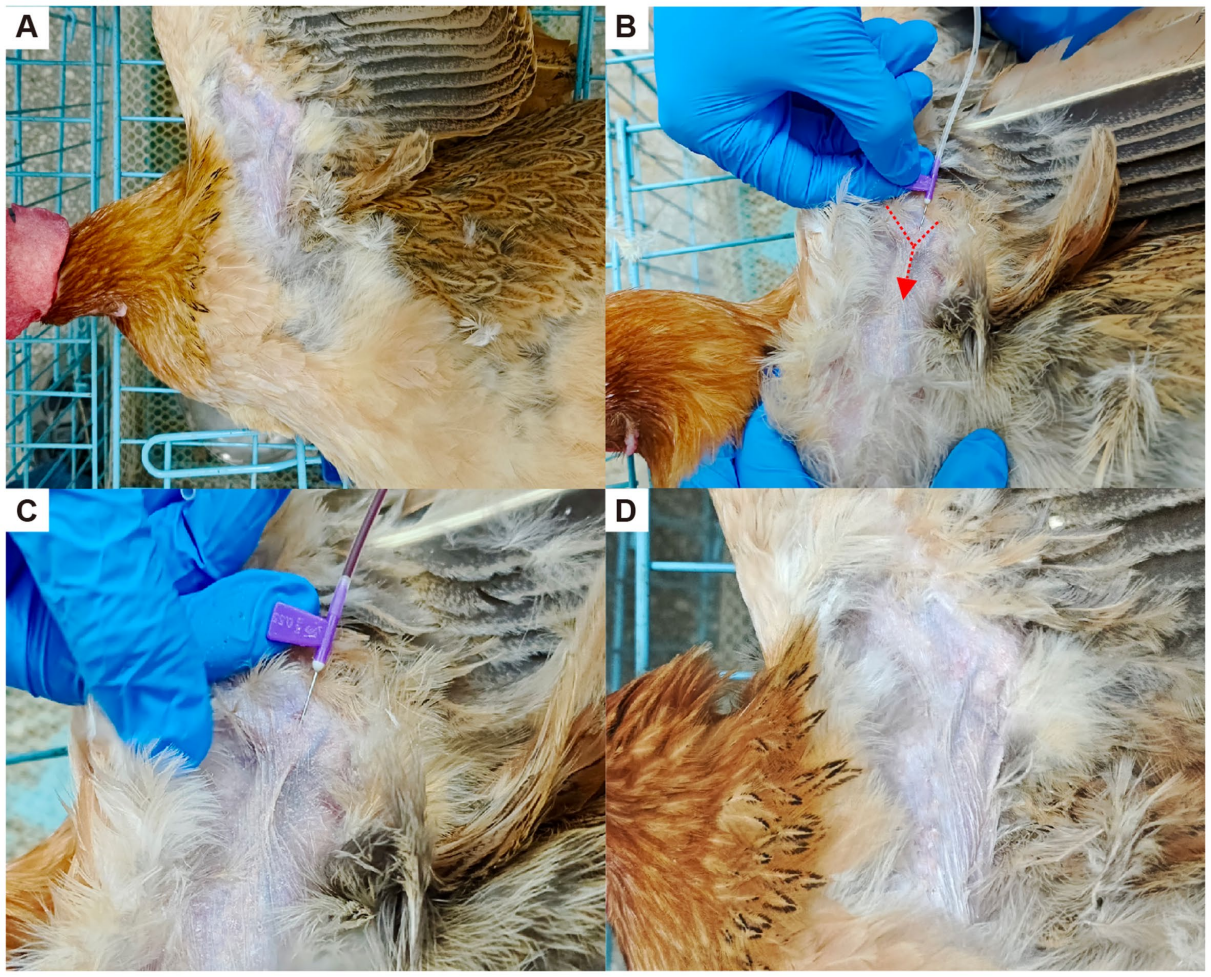
Diagram of the blood collection technique from brachial wing vein in broilers. **(A)** Bird restraint and feather removal around the brachial venous fossa. **(B)** Needle insertion along the course of the vein in the direction of blood flow. **(C)** Blood collection with the needle kept in place, requiring only gentle fixation of the needle. **(D)** Appearance of the sampling site after completion of blood collection, with no obvious visual obstruction at the puncture area. Arrows indicate the centripetal direction of needle insertion.

It is worth mentioning that in pharmacokinetic studies, the blood collection sites include the jugular vein, the brachial (wing) vein and the heart (40). Considering the convenience of restraint and better animal adaptability, brachial wing vein location is generally preferred for repeated sampling, and blood is usually collected via an indwelling catheter or direct venipuncture (41, 42). However, when plasma concentrations of the test substance decrease over time, contamination of the catheter or needle can affect drug concentrations in subsequent samples. In addition, ligation and collapse of the cannulated vein after catheter removal often lead to hematoma formation, which can obscure the brachial vein and complicate further sampling (43). To satisfy the need for serial blood sampling, we adopted an excellent blood collection method–Venous Fossa Undulating Needle Insertion (which we call the “Delta Insertion” blood collection method). This was done by inserting the needle into the posterior edge of the tendon in a proximal direction at a 25° to 30° angle along the junction of the elbow joint veins in the forearm (as shown in Figure 9). Then, puncturing the skin and maintaining negative pressure, the tip of the needle penetrated the underlying muscle and slowly raised to the lowest point of the cardinal venous fossa ventral to the needle until the rupture of the vessel was felt. This method leads to faster and more efficient bleeding than the other two locations (previously mentioned) methods of blood collection. Primarily attributed to the superior muscle compression capabilities, the technique benefits easier hemostasis following needle removal, reduces the likelihood of hematoma formation, minimizes stress on chickens, and better satisfies the blood collection requirements for pharmacokinetic studies. We advocate for broader technical dissemination among veterinary professionals to ensure accurate and efficient blood sample collection while upholding animal welfare standards.

Many drugs, including doxycycline, have been reported to show unusual pharmacokinetic behavior by producing a significant secondary peak in the plasma concentration-time curve after oral administration. (Figure 8) (44, 45). The results of one study showed that after a single oral dose of 20 mg/kg doxycycline hyclate soluble powder, the *t*_1/2_ was 7.78 h and the AUC_0-∞_ was 94.19 h·μg/mL in 30-40-day-old broilers, while the *t*_1/2_ was 19.90 h and the AUC_0-∞_was 121.08 h·μg/mL after multiple oral doses (46). The elimination of doxycycline was significantly slower after multiple oral doses of doxycycline. Reference product 2 in this study was administered in the same form and dose as in that study, with a *t*_1/2_ of 6.16 h and an AUC_0-∞_ of 80.94 h·μg/mL, which was close to the results of that study. In the present study, the *t*_1/2_ (5.92 h) was shorter than that of quails, ducks, geese and ostriches (10.98, 17.65, 13.35, and 19.25 h, respectively) after a single oral dose of 20 mg/kg in broilers. The T_max_ of broilers (3.47 h) was later than that of laying hens, quails, ducks, geese and ostriches (1.73, 2, 2, 2, 2, and 3.03 h, respectively). At the same oral dose, the C_max_ (7.465 μg/mL) was close to that of laying hens and geese (5.88 μg/mL and 6.67 μg/mL, respectively) but lower than that of ducks and quails (17.57 μg/mL and 15.33 μg/mL, respectively) and higher than that of ostriches (0.3 μg/mL, 15 mg/kg) (47–52). The above differences in results may be due to differences in animal species and routes of administration. Recent population pharmacokinetic modelling of doxycycline administered via drinking water in chickens has also highlighted considerable between-bird variability in systemic exposure, especially under field-relevant infection conditions (53).

Before drug administration, a fasting protocol was recommended to minimize experimental variability. Our preliminary work showed that inadequate fasting led to irregular pharmacokinetic profiles, and previous studies have demonstrated modest effects of feeding status on doxycycline bioavailability and T_max_ in chickens. Using the same route and dose of administration, P. Laczay et al. found that the bioavailability was found to be 73.38 ± 2.47% and 61.07 ± 4.39% in fasted and non-fasted chickens, with a C_max_ of 4.47 ± 0.16 μg/mL and 3.07 ± 0.23 μg/mL, respectively. The T_max_ was 1.73 ± 0.06 h and 3.34 ± 0.21 h, respectively (54). Unsurprisingly, the bioavailability of doxycycline is less affected by food, but the presence of food does slow down the absorption of doxycycline to a certain extent.

Unlike traditional bioequivalence studies that typically compare a test product with a single reference formulation, this study involved two reference products, namely a commercial oral solution and a soluble powder. This design was chosen because no approved doxycycline oral solution is currently marketed for poultry in mainland China, where drinking-water administration is predominantly achieved via soluble powders. Furthermore, our research indicated that propylene glycol was essential for the oral solution system. However, it was unclear whether propylene glycol would affect the pharmacokinetic characteristics of DOX in broilers. Our results showed that the test product and both reference products had similar pharmacokinetic profiles in broilers, with no statistically significant differences in log-transformed C_max_, AUC_0-t_ and AUC_0-∞_ (*p* > 0.05). The 90% confidence intervals of the geometric mean ratios for these parameters were all within 80–125% (55), indicating bioequivalence and supporting the clinical interchangeability of the 30% DOX·HCl solution with existing commercial products.

This study also has several limitations. First, dilution stability and mixing behavior were evaluated using a single simulated livestock drinking-water condition, which may not fully represent the diverse water qualities, delivery systems and management practices encountered on commercial farms. Second, the pharmacokinetic and bioequivalence assessments were carried out in healthy broilers under controlled experimental conditions, and clinical efficacy, safety and ease of use were not evaluated in naturally infected or large-scale commercial flocks. Third, only a single oral dose level and regimen (20 mg/kg.b.w.) was investigated, and no formal pharmacokinetic/pharmacodynamic (PK/PD) analysis was performed. Future work should therefore assess the performance of this 30% DOX·HCl solution under different farm water qualities and dosing systems, integrate PK/PD modelling with clinical outcome studies in diseased flocks, and explore its potential application in other important food-animal species. In addition, combination strategies that enhance doxycycline activity against biofilm-forming pathogens, such as co-administration with N-acetyl-L-cysteine, have shown promising *in vitro* results (56), and could be explored in future formulations or dosing regimens.

## 5 Conclusion

In summary, we successfully developed a 30% (w/v) doxycycline hyclate drinking-water solution using propylene glycol as the sole solvent and established an industrially feasible preparation process. The formulation showed good dilution behavior and stability in simulated livestock drinking water, as well as favorable long-term stability under standard storage conditions. Bioequivalence study in broilers demonstrated that the 30% DOX·HCl solution is bioequivalent to two commercially available doxycycline products administered via the same route. Together, these findings indicate that the DOX·HCl solution is suitable for clinical use and provides an accurate, convenient and efficient option for mixed drinking-water medication in livestock and poultry.

## Data Availability

The original contributions presented in the study are included in the article/Supplementary material, further inquiries can be directed to the corresponding author/s.
