# Supplementary material for: A high content (30%) stable doxycycline hyclate drinking-water solution for broilers: preparation, stability, and bioequivalence
Source: Front Vet Sci. 2026 Jan 7;12:1740059. doi: 10.3389/fvets.2025.1740059 (PMC12819747; doi:10.3389/fvets.2025.1740059)
Supplement: Supplementary file 1 [file Table_1.DOCX]

Supplementary Table 1 Stability results of doxycycline in chicken plasma samples (Mean ± SD, n = 6)

| Treatment | Labeled values | Measured values | CV/% |
| --- | --- | --- | --- |
|  |  |  |  |
| Repeated freeze-thaw cycles for 3 times | 0.75 µg/mL | 0.82 ± 0.03 | 3.84 |
|  | 8.00 µg/mL | 8.08 ± 0.23 | 2.81 |
| Untreated samples placed at room temperature for 5 h | 0.75 µg/mL | 0.80 ± 0.05 | 6.06 |
|  | 8.00 µg/mL | 7.38 ± 0.08 | 1.12 |
| Placed in autosampler (26 ℃) for 24 hours | 0.75 µg/mL | 0.77 ± 0.02 | 1.98 |
|  | 8.00 µg/mL | 7.41 ± 0.06 | 0.85 |
| Stored in a -20 °C refrigerator for 3 months | 0.75 µg/mL | 0.84 ± 0.02 | 2.8 |
|  | 8.00 µg/mL | 8.17 ± 0.21 | 2.57 |

Supplementary Table 2 Pharmacokinetic parameters bilateral t-test results.

| Items | Parameters | t1_TOST | t2_TOST | prob_80_00 | prob_125_00 |
| --- | --- | --- | --- | --- | --- |
| Test product and Reference product 1 | Ln（C_max_） | 2.856 | -3.208 | 2.820E-03 | 0.001 |
|  | Ln（AUC_0-t_） | 3.188 | -2.778 | 1.070E-03 | 0.004 |
|  | Ln（AUC_0-∞_） | 3.283 | -2.782 | 0.802E-03 | 0.004 |
| Test product and Reference product 2 | Ln（C_max_） | 4.644 | -1.991 | 7.780E-06 | 0.025 |
|  | Ln（AUC_0-t_） | 4.264 | -1.787 | 3.087E-05 | 0.039 |
|  | Ln（AUC_0-∞_） | 4.276 | -1.749 | 2.954E-05 | 0.042 |
